# Health service access for ethnically underrepresented communities: A scoping review of complex interventions

**DOI:** 10.1371/journal.pone.0340079

**Published:** 2026-01-06

**Authors:** Jessica Rose Pawson, Simon Cannon, Saira Nazeer, Caroline Phillpott, Tadala Kolawole, Joanna Fletcher, Alison Thomson, Jamie Ross, Katie Jane Sheehan, Gillian Woolhead

**Affiliations:** 1 Bone & Joint Health, Blizard Institute, Queen Mary University of London; 2 Therapies department, Barts Health NHS Trust; 3 Orthopaedic Research department, Barts Health NHS Trust; 4 Wolfson Institute of Population Health, Queen Mary University of London; 5 Department of Health Sciences, Brunel University; University of Coimbra: Universidade de Coimbra, PORTUGAL

## Abstract

**Background:**

The aim of this scoping review is to systematically map the existing evidence of complex interventions used to improve access and/or engagement of ethnically underrepresented communities with healthcare services in the UK, and explore equity factors of the evidence.

**Methods:**

The review was guided by the Preferred Reporting Items for Systematic Reviews and Meta-Analyses Extension for Scoping Reviews Checklist. Six databases were searched. Analysis was completed using reflexive and thematic content analysis. Interventions were mapped against the five domains of the Patient-centred access framework and the PROGRESS-Plus equity framework.

**Results:**

A total of 35 studies met the inclusion criteria. Of these studies 63% were completed in primary care, 31% in mental health, 3% in maternity services and 3% in dental care. Several communities were represented, including Gypsy, Roma and Travellers, African Caribbeans, Jewish communities, as well as broader groups such as South Asians, migrants and Muslims. Complex interventions were spread across the five domains of the patient centred access framework and included strategies such as outreach workers, using community settings such as places of worship, cultural adaptations and cultural training for healthcare staff. Effectiveness of interventions was assessed in a third of studies. PROGRESS-Plus analysis identified exclusion criteria including age groups (e.g., below 18 years), ethnic groups (e.g., not first-generation migrants), disabilities (e.g., severe mental health problems) and plus (e.g., language proficiency).

**Conclusions:**

All domains of the patient centred access framework were represented with approachability and acceptability being the most common. Effectiveness assessment was not included in two thirds of studies however, complex interventions show promise to improve equity in ethnically underrepresented communities. Future research should endeavour to assess effectiveness and the translation of interventions in healthcare contexts outside of primary care and mental health. Future policy and research should consider how to increase transparency of power and build strong collaborations with underrepresented communities.

## Introduction

In the United Kingdom (UK), the pursuit of equitable access and improved health outcomes remains a paramount goal [1,2]. Despite progress, significant disparities persist, particularly in ethnically underrepresented communities and low socioeconomic status groups, where life expectancy gaps reach 10.3 years in men and 8.3 years in women compared to high socioeconomic status White British counterparts [1,2]. Indeed, UK ethnically underrepresented communities experience higher rates of long-term conditions [2], poorer healthcare access [2–4], and poorer health outcomes [2,5].

Disparities in healthcare access among ethnically underrepresented communities arise for several reasons. For example, power, shame and cultural influences were found to influence health-seeking behaviours among ethnically underrepresented communities suffering from psychosis [6,7]. Studies in palliative and end-of life care in Gypsy, Traveller and Roma communities identified insensitivity in health services regarding community involvement in decisions and cultural death rituals fostering mistrust in healthcare systems [8,9]. A systematic review exploring bereavement across ethnically underrepresented communities also identified lack of staff cultural awareness across Bangladeshi, Black Caribbean and Gypsy and Traveller communities [10]. An Australian review exploring patient safety found people from ethnically underrepresented communities had higher rates of hospital acquired infections, adverse drug events, complications, and dosing errors when compared to the wider population [11]. It identified contributing factors to increased risk of safety events included language proficiency, beliefs about illness and treatment, formal and informal interpreter use, consumer engagement, and interactions with health professionals [11].To address disparities in healthcare access, multifaceted solutions often characterised as complex interventions are required [1].

A scoping review of non-UK healthcare studies identified inclusion strategies such as culturally and/or linguistically matched staff, identifying uninsured individuals, communication strategies such as specific phone numbers and streamlining services by creating single points of contact [12]. Studies outside of the UK provide critical background with shared barriers to healthcare such as language proficiency, health literacy, valuing traditional medicine and modesty existing across different groups [13]. However, ultimately the healthcare system context shapes access and delivery of care and this varies between public systems such as the UK and private and insurance-based health services.

### Aim

The aim of this review is to synthesise the evidence for complex interventions to support access and/or engagement of ethnically underrepresented communities in UK health services. The synthesis will align to Levesque et al.’s patient-centred access framework which encapsulates five service domains: approachability, acceptability, availability, affordability, and appropriateness [14]. Data extraction and analysis will also include equity factors guided by the PROGRESS-PLUS framework [15]. The findings will enable 1) the identification of gaps for future research and 2) preliminary guidance for policymakers, clinicians and public health bodies on how best to support healthcare service access and engagement of ethnically underrepresented communities.

## Methods

This review is reported according to the Preferred Reporting for Systematic Reviews and Meta-Analysis extension for Scoping Reviews (PRISMA-Scr) checklist [16].

### Eligibility criteria

Studies of interventions to improve ethnically underrepresented communities access to and/or engagement with adult UK healthcare services were included. These included interventions targeted at both patients and healthcare professionals (e.g., cultural competency training). The UK Medical Research Council (MRC) define complex interventions as having several interacting components and/or the properties itself being complex [17]. The MRC also describe how complex interventions may require flexibility or permit tailoring, be dependent on the behaviours of those delivering and receiving the intervention and involving multiple systems (e.g., individual, organisational, societal) [17]. This definition has been applied to the studies within this review. Ethnically underrepresented communities were defined as all ethnic groups except White British [18]. Studies focused on testing existing healthcare interventions on ethnically underrepresented communities but not aiming to improve access and/or engagement in health services were excluded. Studies exploring underrepresented groups where ethnically underrepresented communities were not > 50% of the participants were also excluded.

### Searches

CINAHL Ultimate, Medline, Psychology and Behavioral Sciences Collection, APA Psych Info, Cochrane Library and Pedro were searched on 8^th^ April 2024. The search strategy and keywords were developed using the Population (ethnically underrepresented communities), Concept (access/engagement), Context (National Health Service [NHS]/UK health services) framework (S1 Appendix) [19]. Searches were limited to English language and by publication date (01/01/2014–08/04/2024). The search was limited to studies published from 2014 onwards to ensure relevance to the current NHS context, as earlier studies may not reflect current policy, service delivery changes or population needs; this timeframe also limited the number of results which aligned with the available resources for this project.

The initial searches were completed on CINAHL and Medline exploring combinations of search terms to identify the most effective terminology before being completed on the remaining databases. Reference and citation lists of included articles were also searched.

### Selection

All search results were exported to the Rayyan database for de-duplication, screening, and selection against eligibility criteria by two independent researchers (JRP, SC, SN, CP, TK) [20]. Conflicts were resolved by consensus.

### Data charting items and process

Data from the included articles was extracted to a standardised Excel data chart by an individual researcher (JRP) and 10% of the data was independently charted by another researcher for comparison (SN) [21]. Extracted data included study title, author, year of publication, study design, location, population, healthcare setting and intervention (S2 Appendix). The intervention components were mapped to the Patient Centred Access Framework [14].Eligibility criteria, baseline characteristics and subgroup analyses were mapped to the Cochrane and Campbell Collaboration Equity methods group’s PROGRESS-Plus framework [15,22]. **PROGRESS** is an acronym for **P**lace of residence, **R**ace/ethnicity/language/culture, **O**ccupation, **G**ender, **R**eligion, **E**ducation, **S**ocioeconomic status and **S**ocial Capital [15,22]. **PLUS** enables the framework to capture other factors which impact equity, such as 1) age 2) disability 3) features of relationships and 4) time-dependent relationships [15,22]. Using the PROGRESS-Plus framework in the inclusion/exclusion analysis provided a structured and transparent way to map equity characteristics of each study, allowing a better understanding of the diversity of the populations represented under the broad term “ethnically underrepresented communities”. This approach helped identify which specific dimensions of disadvantage (e.g., language, socioeconomic position, language) were considered across the studies, highlighting variation and gaps in how equity has been defined and operationalised.

### Synthesis of results

Numerical content analysis was used to summarise study designs, locations, populations and healthcare settings. The remaining data analysis was guided by Braun and Clarke’s [23] Thematic Content Analysis and reflexive thematic analysis [24]. Analysis involved deductively categorising components of interventions to Levesque et al’s patient-centred access framework domains [14]. The software Trello was used to extract and code the themes of each domain into categories (combining similar themes or those with overlaps). Trello was utilised to support organisation, visualisation and coding of themes, whilst it is typically an organisational tool, it has also been used in other qualitative studies to support reflexive thematic analysis [25]. The included papers were re-read to inductively assess for any additional themes not encompassed in the patient-centred access framework. After the information on Trello was finalised, a Word document of themes and categories was then developed and reviewed (by JRP and GW) (S4 Appendix).

## Results

**Fig 1:**
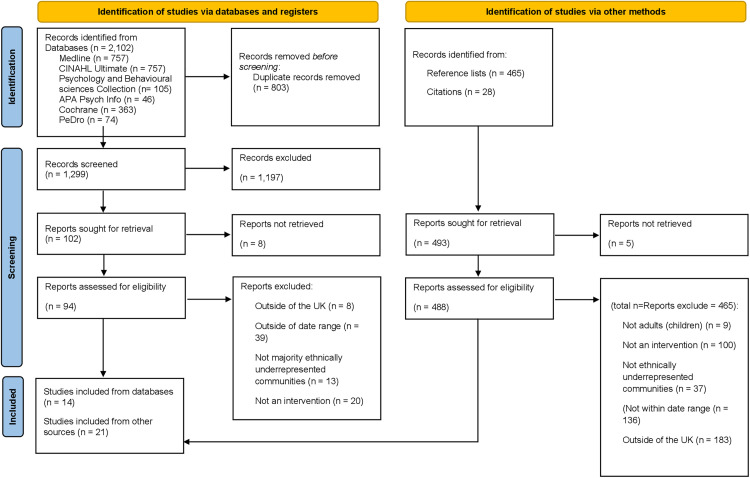
PRISMA diagram [26].

The search on CINAHL Ultimate retrieved 757 results, Medline retrieved 757, Psychology and Behavioural Sciences Collection retrieved 105, APA Psych Info retrieved 46, Cochrane Library retrieved 363 and Pedro (The Physiotherapy Evidence Database) retrieved 74. This identified a total of 2,102 results from databases. Duplicates were removed in Rayyan, resulting in a total of 1,299 articles. Reference lists of included articles were also searched resulting in 465 more articles being assessed for eligibility and citations sourced another 28 articles. After screening against the eligibility criteria, 35 studies were included in this scoping review, 14 from databases and 21 from other sources (reference and citation tracking) (Fig 1).

Included articles were published between 2014 and 2024. Interventions included (but not limited to) outreach workers employed to target ethnically underrepresented communities for health checks [27–29], a family based culturally adapted intervention for schizophrenia [30,31] and physical activity and lifestyle interventions [32,33]. Table 1 includes a full summary of study and intervention characteristics.

**Table 1:** Summary of included studies.

Citation	Study design	Study aim	Health service	Population	Area of the UK	Intervention
Dyson, 2020 [34]	Mixed Methods: Intervention Mapping, prioritisation, cross community synthesis of interventions	The study aims were to a) Investigate facilitators and barriers to acceptability and uptake of immunisations amongst six Gypsy, Roma and Traveller communities across four UK cities and b) to identify potential interventions aimed at increasing uptake of immunisations in these communities.	Primary care (immunisation)	51 Gypsy, Roma and Travellers and 21 service providers	Bristol, York, Glasgow and London	This priority setting exercise of potential interventions included: healthcare professional cultural competence training, ways to identify Gypsy, Roma and Travellers in Health records to monitor uptake and tailor support, allocating a named frontline person in GP Practice, having flexible and diverse appointment booking, recall and reminder systems, and protected funding for specialist health visitor (community outreach)
Edge, 2016 [30]	Protocol for a mixed methods feasibility cohort design study	This protocol outlines the study plans which are to test the feasibility and acceptability of delivering Culturally-adapted Family Intervention (CaFI) to African-Caribbean service users diagnosed with schizophrenia.	Community Mental Health	African-Caribbeans diagnosed with schizophrenia and their families (protocol)	Manchester	CaFI is a culturally adapted version of a structured, cognitive-behavioural model of Family Intervention comprising psycho-education, cognitive behavioural skills for stress management, coping and problem solving. Key aspects of the culturally adapted version include focusing on recovery and adopting a shared learning approach where therapists play to family strengths.
Edge 2018 [31]	Mixed methods study including intervention development, evidence synthesis, feasibility and acceptability testing	This study aims to test the feasibility and acceptability of delivering Culturally-adapted Family Intervention (CaFI) to African-Caribbean service users diagnosed with schizophrenia.	Community Mental Health	31 African -Caribbeans diagnosed with schizophrenia and their families	Manchester	Same as intervention above
Goff, 2021 [33]	Mixed methods randomised controlled feasibility trial	Study aimed to evaluate the acceptability, fidelity and trial feasibility of the culturally tailored type two diabetes self-management education and support program in Black British adults – the Healthy Eating and Active Lifestyles for Diabetes (‘HEAL-D’)	Primary care (Diabetes)	63 Black British Adults	London (boroughs of Lambeth and Southwark)	Intervention. The Healthy Eating and Active Lifestyles for Diabetes (“HEAL-D”) intervention developed as per Moore et al. (2019).
Penn, 2014 [35]	Qualitative study using the Theoretical Domains Framework	To investigate Pakistani female participants’ perspectives of their behaviour change and of salient intervention features	Primary care	20 Pakistani females	Middlesborough	New Life, New You. Culturally adapted behavioural change programme aimed to improve Physical Activity levels in ethnic minorities. A community based 8 weeks programme of group based advice.
Latif, 2016 [36]	Pilot study (Pre-test, post-test)	To assess the effectiveness of a health promotion video delivered in Bengali (native language to the local community population) in a community setting for coronary artery disease.	Primary care (health promotion)	18 Bangladeshi (eighteen females)	South London	An educational video was developed using the British heart foundation guidance and Bengali cultural and language adaptations, on the signs, symptoms and prevention of coronary artery disease. The video was played in Bengali to a Bangladeshi women’s group.
Such, 2019 [32]	Analysis of secondary dataset	Analysis of a physical activity data set of programmes focussed on minority ethnic populations.	Primary care (health promotion)	57 programmes focusing on minority groups broadly	UK wide dataset	Three case studies used in the paper to demonstrate features of the physical activity interventions included in the dataset. Interventions included for example: community venues, multilingual facilitators, factoring in cultural beliefs and values to support programme design and delivery.
Yuan, 2019 [37]	Quasi-experimental study without randomization	The study aimed to evaluate a culturally appropriate community-based oral health education intervention.	Dental Health	17 Chinese “newly arrived” undocumented migrant mothers of infants	Northern Ireland	Culturally tailored community education through telephone and home visits and unlimited social support. Oral health intervention was delivered over 12 months and focused on oral health related knowledge, attitudes and behaviours, including knowledge of tooth brushing, sugar consumption and other oral related behaviours.
Khan, 2019 [38]	Mixed methods feasibility study: qualitative and pre-test, post-test feasibility and acceptability	This study aims to assess the acceptability and feasibility of a cognitive behavioural therapy-based intervention called the Positive Health Program (PHP) in British Pakistani women. The study also assessed experiences of depression and the intervention.	Maternal Mental Health	36 British Pakistani Women	Manchester	Positive Health Programme (PHP) – Culturally adapted psychological group based 12 week intervention. Elements include: delivery focused on maternal well being and not just postnatal depression, community based programme and identification and inclusion of the specific pressures and expectations of being a British Pakistani woman.
Stone, 2019 [27]	Qualitative interview study of 15 members of staff	To understand staff perspectives of a novel telephone outreach intervention that aims to improve the uptake of NHS Health Checks in Black, Asian and Ethnic Minority communities where the risk of cardiovascular disease is higher.	Primary care	Black, Asian and Ethnic minority groups	Bristol	Novel telephone outreach using Telephone Outreach Workers that contact patients from minority ethnic groups to inform them they are eligible for a health check and support booking them an appointment.
Filby, 2020 [39]	Service evaluation	To evaluate a specialist migrant maternity service	Maternal care	10 Migrant mothers of different nationalities	Kings College Hospital, London	Antenatal maternity care provided within the initial accommodation centre and supported transport to hospital appointments, supported women regarding healthcare subsidies and charges, worked collaboratively with other relevant services (such as GPs, health visitors, Refugee Council). Founded the multilingual baby group the South London Happy Baby Group Community.
Mir, 2015 [40]	Mixed Methods: Qualitative interviews and feasibility and acceptability of the developed intervention	To develop cultural adaptations of the existing behavioural activation manual and to assess the feasibility and acceptability. It is anticipated that this adaptation may help facilitate effective relationships between Muslim patients and therapists to support therapists to respectfully engage patients about their religious identity.	Community mental health	19 self-identifying Muslims	Bradford, UK	Culturally adapted Behavioural Activation (BA) for depression. BA was adapted to add the discussion of environmental stressors in relation to depression and this link to religion, patients were offered a choice of culturally matched therapists or not (due to mixed findings), therapists were educated about Islam, and the adaptation promoted professionalism and a non-judgemental approach to these conversations). Therapists assessed how important religion was to patients and used this to guide the BA focus. A self help guide was also developed Between 6 and 12 sessions were offered and family were offered to be involved.
Moore, 2019 [41]	Intervention development using focus groups with patients	The aim of this study was to develop a culturally sensitive intervention for type two diabetes mellitus for people of African Caribbean ethnicity, using the Behaviour Change Wheel and associated COM-B methodological framework to identify relevant behaviour change techniques.	Primary care (Diabetes)	41 Adults of self-declared Black African (BA) or Caribbean (BC) ethnicity with a clinical diagnosis of type two diabetes mellitus	South London, UK	The Healthy Eating and Active Lifestyles for Diabetes (“HEAL-D”) intervention involved: information about health consequences and benefits of target behaviours including self-management, social support and social comparison (sharing success and failures), demonstration, training and instruction on how to perform behaviours, graded exercise tasks, credible sources and social comparison (development of videos featuring patients and professionals), goal setting and feedback on outcomes of behaviour and action planning. Intervention tested by Goff et al. (2021).
Masood, 2015 [42]	Nested qualitative study, part of an exploratory randomised controlled trial (RCT) conducted to test the feasibility and acceptability (study by Khan et al., 2019)	The aim of this study was to assess the acceptability and overall experience of the Positive Health Programme by British South Asian mothers.	Community mental health (postnatal)	83 British South Asian women with postnatal depression	Lancashire and Manchester	Positive Health Programme (PHP) – Culturally adapted psychological group based 12 week intervention. Elements include: delivery focused on maternal well being and not just postnatal depression, community based programme and identification and inclusion of the specific pressures and expectations of being a British Pakistani woman.
Husain, 2023 [43]	Two-arm single-blind exploratory randomised controlled trial	To test the feasibility, acceptability and the efficacy of ROSHNI-D, a culturally sensitive group CBT intervention, the Positive Health Programme (PHP) in British South Asian women with Postnatal depression and compared it to a routine treatment group	Maternal mental health	83 British South Asians Adults with maternal depression	Lancashire and Manchester	Positive Health Programme (PHP) – Culturally adapted psychological group based 12 week intervention. Elements include: delivery focused on maternal well being and not just postnatal depression, community based programme and identification and inclusion of the specific pressures and expectations of being a British Pakistani woman.
Buffin, 2015 [28]	Mixed methods – survey and qualitative interviews	To describe a peer outreach initiative used to increase organ donation in ethnic minorities	Primary care (organ donation/kidney)	800 participants – 70% Asian, 19.7% Black African/African Caribbean	London	Peer outreach program at community events aimed to increase organ donors. Examples of the type of events attended by the Peer Educators include an Asian bridal show, a public library and a yoga class for South Asian elders.
Kelly, 2020 [44]	Prospective feasibility study	(1) The feasibility of recruiting South Asian migrants to view an educational film on Chronic Viral Hepatitis (CVH), [2] the effectiveness of the film in promoting testing and increasing knowledge of CVH, and [3] the methodological issues relevant to scale-up to a randomized controlled trial	Primary care (infectious diseases)	219 participants recruited of South Asian heritage	South East England	Educational film with information about testing for Hepatitis was developed using focus groups and inclusion of personal stories of positive and negative effects of testing. Video is here: https://youtu.be/K3AYyZ3uHro
Jain, 2014 [29]	Descriptive article	To describe the work of Kidney Research UK Charity in relation to patient and public engagement, describing the Peer Educator model and providing examples including the “end-of-life project”, the deployment and impact of Peer Educators	Primary care (kidney research)	The end-of-life project reached ~2,700 beneficiaries across 4 sites	UK broadly (Bedfordshire and 4 other renal units)	Peer educators completing patient engagement and outreach activities
Griffiths, 2016 [45]	Cluster randomised control trial	To test the hypothesis that a culturally specific education programme, adapted from promising theory-based interventions developed in the USA, would reduce unscheduled care for South Asians with asthma in the UK	Primary care (asthma)	375 total (183 intervention group and 192 in control group)	London boroughs of Tower Hamlets and Newham	The Physician Asthma Care Education (PACE) programme and the Chronic Disease Self-Management Program (CDSMP) was adapted for use with South Asians (however, limited information was provided on how it was adapted)
Bush, 2014 [46]	Cluster randomised control trial	This proof-of-concept study aimed to test this hypothesis using a cluster randomised controlled trial (RCT); to our knowledge, this is a novel study aimed at increasing uptake of retinopathy screening in a hard-to-reach and high-risk population group	Primary care (diabetes)	Number of South Asians contacted and screened not reported	Coventry	Link workers telephoning patients that fail to attend screening appointments – minimal details provided about the link worker’s backgrounds/linguistic skills etc.
Crawshaw, 2023 [47]	Mixed methods: Qualitative and co-design	To construct a community‐based participatory research (CBPR) study with Congolese migrants in the United Kingdom to understand the complex mechanisms influencing their COVID‐19 vaccination attitudes, beliefs and behaviours, and use behavioural theory and participatory co‐design methods to translate these findings into a tailored intervention to strengthen their COVID‐19 vaccine uptake	Primary care/public health	32 participants in the qualitative interviews and 16 in the co-design workshops (2x 8)	London borough of Hackney	Plan/components developed using CBRP - Intervention component 1: Community‐led workshops (opportunities to have two way conversations about vaccines, sessions to be hosted by local community organisations with specialists and healthcare professionals as guests), intervention component 2: Short plays (use of storyboards to tell stories about vaccines using humour and cultural references), Intervention component 3: Posters and flyers (again using cultural references, appropriate colours and images to convey messages) – other components were identified by the groups as potential for behaviour change opportunities
Offman, 2014 [48]	Observational	To report on a telephone reminder intervention in Newham taking place in the context of the 2010 round of screening, in the NHS Breast Screening Programme	Primary care/public health	Women living in Newham that were eligible for breast screening. Used aggregate GP data so does not provide number of participants	London borough of Newham	A charity (Community Links) in Newham was commissioned to complete telephone intervention invitation. Local women understanding the socio-cultural profile of the community in Newham and speaking combinations of the following languages spoken in Newham were recruited: Hindi, Urdu, Punjabi, Gujarati, Bengali, Somalia, French, Spanish and English. They attended one training programme covering a general overview on breast cancer and breast cancer screening, the benefits and risks of screening, informed consent and information governance
Roche, 2018 [49]	Mixed methods: Qualitative study and feasibility cluster randomised controlled trial	To [1] develop a new intervention to facilitate timely diagnosis of dementia in Black African and Caribbean populations, and [2] evaluated its acceptability and [3] its delivery in a feasibility cluster randomised controlled trial (RCT)	Primary care/elderly care	47 participants of Black African, Black Caribbean or Black British heritage	London – (5x London boroughs)	Leaflet entitled “Getting help for forgetfulness” with accompanying letter from GP practice reassuring that the letter wasn’t based on information from GP but that it included advice about forgetfulness and when/how to seek help
Parveen, 2017 [50]	Qualitative (focus groups)	The objectives were to establish whether Alzheimer’s Society Information Programme for South Asian families (IPSAF) had an immediate and medium‐term impact on those who attended and how it impacted on the wider family and the person with dementia	Primary care/elderly care	42 participants of Indian, Pakistani, and Bangladeshi heritage	7 locations across the UK	IPSAF is a culturally adapted programme for people with dementia and their family/carers. Aiming to improve the knowledge, skills, and understanding of South Asian carers supporting a relative with dementia. The programme is delivered by an Alzheimer’s Society facilitator in partnership with a local South Asian community organisation and consists of 4 sessions addressing understanding dementia, legal and money matters, looking after others, and looking after yourself.
Robinson, 2022 [51]	Co-design workshops	Through codesign workshops, this study seeks to integrate the voices of those people from ethnic minority populations to gain better insight and create recommendations, on improving access to medicines advice from community pharmacies for people from ethnic minority communities	Primary care (pharmacy)	12 participants belonging to ethnic minority groups (non White British) taking one or more regular prescription medicine	North of England	Codesign workshops developed 3x recommendations: Recommendation 1: Delivering and providing culturally competent medicines advice. Recommendation 2: Building awareness of accessing medicines advice from community pharmacies. Recommendation 3: Enabling better discussions with patients from ethnic minority communities
Lamb, 2015 [52]	Qualitative evaluation	To develop and evaluate a model for the community engagement component of the complex intervention, focusing on developing relationships with stakeholders, their engagement with the issue of access to mental health and with the programme through the Community Engagement model.	Community mental health	4x community focus groups with 15, 11, 25,17 participants but no further information about participants	Longsight, Greater Manchester	Mental Health Champions were engaged to undertake targeted outreach within the local community; deliver 2-hourly psychological interventions linked with mild–moderate physical activities over six weeks – delivered by mental health practitioners and co-facilitated by an expert patient; and on-going monitoring and evaluation of the project (limited other details)
Lwembe, 2017 [53]	Qualitative	This study seeks to build an understanding of whether and how lay contributions from community and well-being champion (CWBC) have impacted on the social determinants of mental health, including the system within and through which the intervention was delivered	Community mental health	25 Black and minority ethnic service users accessing mental health (community services)	West London	Mental Health Champions were engaged to undertake targeted outreach within the local community; deliver 2-hourly psychological interventions linked with mild–moderate physical activities over six weeks – delivered by mental health practitioners and co-facilitated by an expert patient; and on-going monitoring and evaluation of the project
Mantovani, 2017 [54]	Qualitative	This study seeks to build an understanding of whether and how lay contributions from community and well-being champion (CWBC) have impacted on the social determinants of mental health, including the system within and through which the intervention was delivered	Community mental health	13 community champions were recruited to the study	South London	The community and wellbeing champions engaged in formal group work and informal one-to-one conversation (favouring group work as this encouraged engagement in sensitive topics). The CWBCs hosted 40 groups in the duration of the study. CWBCs had different styles of intervention which can be divided into: (i) talking to people informally about mental health as part of their daily lives; (ii) providing more intensive support to individuals; (iii) imparting knowledge on how to take control over one’s own health; and (iv) partaking in or managing/leading activities, groups or events in the associated Faith Based Organisations.
McEvoy, 2017 [55]	Mixed methods	This paper offers a critical reflection upon an initiative that sought to improve access to an NHS funded primary care mental health service to one ‘under-served’ population, an Orthodox Jewish community in the North West of England	Community mental health	12 stakeholders (3 patients) involved in the project	Salford	“Eis Ledader” project (which means ‘time to talk’ in Hebrew) was developed with the aims to reduce the stigma in the Jewish community of seeking help for mental health needs, the project did this through the following overarching aims: establishing an arms length relationship, building a collaborative partnership and building a mature collaborative partnership
Owit, 2014 [56]	Mixed methods – ethnographic and quantitative analysis	This paper reports on the findings that demonstrate the effectiveness of the cultural consultation service (CCS) model in facilitating the learning of cultural competence skills of clinicians within clinical encounters	Community mental health	30 clinicians attended 15 cultural competence training sessions	London borough of Tower Hamlets	Cultural Consultation Service (CCS) – provided cultural competence training and also consulted regarding patients (sat in on sessions) and provided advice about patients
Low, 2024 [57]	Mixed methods service evaluation	This paper presents an evaluation of the feasibility and acceptability of delivering the HEAL-D Online service using an online platform delivered by the National Health Service (NHS) in South London. The evaluation aimed to examine the following factors: (i) acceptability to service users; (ii) feasibility to staff delivering the online programme; (iii) feasibility of digital participation for service users; (iv) potential benefits to service users following participation; (v) potential future improvements to HEAL-D Online.	Primary care (Diabetes)	53 HEAL-D service users completed a post course questionnaire, and 14 service users and 7 service delivery staff participated in interviews	South London	HEAL-D online is comprised of: information about health consequences and benefits of target behaviours including self-management principles, social support and social comparison (sharing success and failures), demonstration, training and instruction on how to perform behaviours, graded exercise tasks, credible sources and social comparison (development of videos featuring patients and HCPs), goal setting (SMART) and feedback on outcomes of behaviour, self-monitoring and action planning
Moore, 2023 [58]	Feasibility (pre-test post-test)	To determine the feasibility of undertaking a community multicultural healthy eating education and cooking intervention featuring traditional African Caribbean foods at a community organization by evaluating service users’ and staff perceptions of the acceptability and relevance of using resources in real life/practice. The second aim was to evaluate the potential impact of the intervention and resources on participant’s food and cooking confidence, knowledge, and behaviours	Primary care (Diabetes)	30 participants consented to attend a workshop and 22 complete the questionnaires	Leeds	1 hour discussion group using resources (East Well guide that had been culturally adapted) and 3 hour cooking session to put the recipes into action
Lowry, 2022 [59]	Protocol of Low et al. (2024)	The purpose of this study is to describe the methods to explore the [1] feasibility and acceptability of a virtual delivery model for HEAL-D in south London and [2] factors affecting its scale-up across other areas in England	Primary care (Diabetes)	Protocol plan to recruit self-identified Black African or Caribbean members of the public with T2DM	South London	HEAL-D online is comprised of: information about health consequences and benefits of target behaviours including self-management principles, social support and social comparison (sharing success and failures), demonstration, training and instruction on how to perform behaviours, graded exercise tasks, credible sources and social comparison (development of videos featuring patients and HCPs), goal setting (SMART) and feedback on outcomes of behaviour, self-monitoring and action planning
Flanagan, 2019 [60]	Cluster randomised control trial	Aimed to determine whether incentivising and supporting primary-care physicians in areas with a high density of migrants increases the numbers of adult migrants screened for viral hepatitis	Primary care	51,773 randomly selected eligible patients in the intervention groups in London and Bradford, letters were sent to 43,585 (84·2%) patients	Bradford, South East and North East London	HepFREE – unclear if this provided any different intervention. GPs in participating sites were given a fee of £500 for signing up and £25 per each participant that they enrolled into the study
Krasuska, 2023 [61]	Stepwise approach intervention development	To use a stepwise approach that was informed by the Six Steps in Quality Intervention Development framework, which consisted of gathering evidence through literature review and focus groups (step 1), developing a program theory for the intervention (step 2), and finally developing the content of the text messages and an accompanying delivery plan (step 3).	Primary care (Diabetes)	25 people took part in 3 focus groups	Scotland	Culturally tailored text messages to prevent T2DM for one specific South Asian population, women of Pakistani origin living in Scotland

### Participants

A total number of 2,761 participants plus 102 stakeholders/service providers were included in the 35 studies. Sample size ranged from three [55] to 800 participants [28]. Four studies did not report the number of participants, two due to the use of aggregate health data [46,48] and two reported on programmes that did not collect numbers of participants they engaged with [29,32]. Studies included different populations including those identifying as Gypsy, Roma and Traveller groups, South Asians, Pakistanis, Chinese migrants, Jewish communities, migrant mothers broadly, self-identifying Muslims, Bangladeshis, Black British people, Black Caribbean or African communities, and Black, Asian and ethnically underrepresented communities more broadly.

### Health services and location

The studies covered various health service areas, with 22 (63%) primary care, 11 (31%) mental health, 1 (3%) dental health and 1 (3%) maternity care.

Geographically, 40% of the studies were in London, 16% in Manchester, 13% were UK wide (or multiple sites across the UK), and the rest distributed between Bradford, Bristol, Coventry, Northern Ireland, Leeds, Middlesborough, North of England, Salford, Scotland and South East England.

### Effectiveness and acceptability of interventions

Effectiveness of interventions was explored by 12 studies [31,36–38,43–45,48,56–58,60]. The studies that assessed effectiveness completed interventions using telephone reminders, GP incentives, culturally tailored programmes for depression, schizophrenia, diabetes, asthma and dental health, educational videos about heart disease and viral hepatitis screening, and a cultural consultation service. Positive results were reported by all studies, with only two studies reporting a neutral effect in some of their measures [44,45]. Table 2 provides further details of interventions and their effectiveness.

**Table 2:** Effectiveness of included interventions.

Intervention	Study design	Population	Effectiveness results
Culturally tailored telephone reminder intervention [48]	Observational study	Ethnic minority women eligible for breast screening	Breast screening uptake was higher in participating practices
HepFREE - GPs in participating sites were given a fee of £500 for signing up and £25 per each participant that they enrolled into the study [60]	Cluster randomised control trial	“Migrants” in participating GP practices	Hepatitis screening uptake was higher in participating practices
The “Positive Health Programme” a culturally tailored intervention for postnatal depression [38]	Feasibility study	South Asian women with postnatal depression	Reduction in depression post intervention
The “Positive Health Programme” a culturally tailored intervention for postnatal depression [43]	Randomised control trial	South Asian women with postnatal depression	Reduction in depression but only a significant difference in women that had attended>four sessions
Culturally tailored video to provide education about coronary artery disease [36]	Pre-test/post-test pilot study	Bangladeshi women attending a community centre	Statistically significant increase in knowledge post intervention
Culturally tailored video to increase awareness of Hepatitis screening [44]	Feasibility study	South Asian migrants	No improvement to knowledge of hepatitis screening
Culturally adapted Family Intervention (CaFI) [31]	Mixed methods feasibility study	African-Caribbean service users diagnosed with schizophrenia	The mean EQ-5D-5L utility index was 0.74 at baseline, rising to 0.82 post intervention. Health utility index scores improved significantly after intervention (mean difference 0.09, 95% CI 0.02 to 0.17; p < 0.05, bootstrapped t-test).
Healthy Eating and Active Lifestyles for Diabetes (‘HEAL-D’) adapted to be delivered online [57]	Mixed methods service evaluation	Black Caribbean, Black African or Black British people with Diabetes	Participants felt more confident in controlling their diabetes and managing their diet post intervention
Cooking intervention using culturally tailored cooking book [58]	Feasibility (pre-test post-test)	Intervention tailored to African Caribbeans, but no ethnic minority groups were excluded from being involved in the study	Familiarity of healthy eating guidance had significantly increased post intervention (pre-intervention 36%, n = 8 to post-intervention 100%, n = 22)
Cultural consultation service (CCS) facilitating the learning of cultural competence skills of clinicians within clinical encounters [56]	Mixed methods	Targeted clinicians working with people from different cultural backgrounds in primary care	Significant improvement in clinician self-rated cultural competence, but they did not assess if this translated to patient outcomes
Culturally tailored intervention to improve dental hygiene in mothers [37]	Quasi-experimental study	Chinese migrant mothers	Improved attitudes to baby teeth brushing
The Physician Asthma Care Education (PACE) programme and the Chronic Disease Self-Management Program (CDSMP) was adapted for use with South Asians [45]	Cluster randomised control trial	South Asians after an asthma exacerbation	Reduction in time to first review, improvement in asthma related quality of life and self-efficacy (at 3 months) but did not show a difference in time to first unscheduled attendance for asthma which was their primary outcome

Acceptability of interventions was explored by five studies [33,38,40,49,58]. The HEAL-D intervention in person [33] and the adapted online version [57] were both well attended and acceptable to Black Caribbean, African and Black British participants. Moore and colleagues [58] found that participants attending their cooking intervention also had high levels of satisfaction. A written and posted information leaflet for dementia awareness in Black African and Caribbean populations was found to have high levels of acceptability (93.6% n = 47) [49]. A culturally adapted behavioural activation programme for depression was found to be acceptable to Muslim participants [40]. Finally, the “Positive Health Programme” was also reported as acceptable to participants in a feasibility study [38].

### PROGRESS-Plus framework

Analysis using the PROGRESS-Plus framework focused on study eligibility criteria (inclusion and exclusion), baseline characteristics and subgroup analyses (S3 Appendix). For eligibility criteria, under **Place of residence** one study excluded care home residents [49], another excluded those living in temporary accommodation [47]. In relation to **Race/ethnicity/culture** two studies excluded people that were not 1^st^ generation South Asian immigrants [36,44], another excluded people that were not 1^st^ generation immigrants from the Democratic Republic of Congo [47] and two studies excluded those not self-identifying as African, Caribbean or Black British [31,49]. **Age** exclusions were utilised by several studies, with seven studies excluding people below 18 years [31,33,36,38,45,51,61], and two studies excluding people below 16 years [42,62], one study excluded those below 25 years [35] and another study those below 50 years old [49]. Many **Disability** exclusion factors were identified, two studies excluded those with severe physical or learning disabilities [38,43], three studies excluded those with severe mental health problems (including depression and psychosis) [33,38,53]. Four studies in community mental health excluded people that were actively suicidal or deemed a high risk to themselves or others [31,38,43,45]. One study excluded those with mobility problems preventing attendance of the programme and “complex clinical needs” [33]. Another excluded those with chronic conditions that would limit engagement in the diet and activity behaviour modification intervention [58]. Finally, two studies excluded people with cognitive impairments, dementia diagnosis or those lacking capacity to consent [31,49]. The **Plus** aspect of the framework identified one study that excluded people that were pregnant and those participating in another research study [33] and four studies excluded patients due to language proficiency [31,44,58,61].

In relation to baseline characteristics, **Place of residence** was reported as “rented, owned or shared” by one study [45]. **Race/ethnicity/culture** was well reported in baseline characteristics with groups as follows, Roma and Traveller communities [34], Black African, Caribbean or Black British [28,31,33,49,54,57,58,60], Asian Caribbean [49], “Asian” [28], South Asian [44,50,51,60], Eastern European [60], White British [51], “Other” [58,60], Arab, Mixed Arab and Turkish [51], Vietnamese, Chinese, Albanian, Nigerian, Afghan, Yemeni [39]. Generation in the UK (1st, 2nd, 3rd, 4th) was reported by two studies [38,45]. Followed by **Occupation**, where four studies reported employment status [31,33,38,49], three studies reported occupations from a list or classification [34,38,50], one study reported if participants were sick, disabled or in receipt of welfare benefits [58].

Three studies included subgroup analyses of their participants [28,45,58]. One study using peer outreach workers to increase organ donation in ethnically underrepresented communities had a higher sign-up rate of Asian people (11%) compared to Black people (2%) [28]. A study delivering an asthma intervention to South Asians [45] found that ethnicity moderated the intervention effect (P = 0.046), “Other South Asians” (Indian, Pakistani or Sri Lankan) had the best outcome (hazard ratio of 1.55), followed by the Bangladeshi group (hazard ratio of 1.11) and the poorest outcomes were seen in the British Bangladeshi group (hazard ratio of 0.72). They found that the role of immigrant generation in the UK also moderated the intervention effect, with first generation immigrants in the intervention group having the best outcomes (hazard ratio of 5.55), followed by first generation immigrants in the control group (hazard ratio of 4.56) and then higher generation immigrants in the intervention group (hazard ratio of 3.12). Finally, they explored the role of English fluency and found weak evidence that this moderated the intervention effect (P = 0.083). One other study looked at a healthy eating and cooking intervention for African Caribbean groups [58] and found those belonging to African Caribbean ethnic groups had a better response to the intervention than those from other groups. They did not find a significant difference in intervention response when comparing educational levels across the whole group and within ethnic groups [58].

### Thematic analysis

This review analysed intervention studies using the five service domains in the patient-centered access framework [14], (S4 Appendix). There was a thorough spread across the five domains of the patient-centred access framework in the complex interventions that were discussed in the articles. Approachability and acceptability had the most supporting sources (30 and 27 respectively). With affordability having the least (16 supporting sources), followed by appropriateness (20 supporting sources) and availability and accommodation (22 supporting sources). The first step of accessing and engaging with a health service is knowing that a particular service exists (approachability) [14]. Eight themes aiming to increase awareness of health services were identified were supported by 30 sources (outreach, education about health services, sign posting to other services, ambassadors/community champions, collaboration with community links, advertisements, screening through health services, communication via phone). The most common activity to increase approachability was outreach to community spaces/organisations (n = 12) [28,32,33,41,42,44,47,51,53–55,58], places of worship (n = 4) [32,33,44,54], an Asian bridal show [28], dementia roadshows [50], community activities such as yoga [28] and support groups [53]. Advertising in Asian supermarkets [32], community centres [33], on the radio [29] and on social media was also used (n = 5) [30–32,51,63]. Community ambassadors/champions were developed by nine studies to form stronger community links and raise awareness [32,33,42,47,52–55,61]. Two studies employed telephone outreach workers for NHS health checks [27,48], while another study utilised outreach workers with Gypsy, Roma, and Traveller communities to improve vaccination uptake [34]. Other studies (n = 9) utilised existing health service structures to identify and contact patients that through General Practitioner/primary care contacts, or those who presented to urgent care or Accident & Emergency departments [27,33,44–46,48–50,60].

The second domain is acceptability [14], which refers to cultural and social factors needed for people to accept services. Thirteen themes were identified supported by 27 sources (cultural adaptations, building trust and relationships, gender split sessions, culturally matched staff, using religious spaces, familiar staff, incorporating social support, activities with minimal social barriers, engagement/information film, content verified by native speakers, consideration of literacy levels, adaptation of language, incorporating cultural and religious values). Religious spaces were utilised to increase cultural and social acceptability of interventions [32,38,41]. Cultural adaptations of programmes were used in nine studies [30,31,38,40,41,45,49,57,58], for example culturally adapted dietary advice to support diabetes, incorporating Caribbean examples instead of Western ones, to enhance relevance and participant adherence [33,41,57]. Another eight studies discussed building trust, therapeutic alliance and relationships [30–32,41,47,50,54,55]. Gender split interventions was considered by three studies [32,54,55], however seven studies targeted women specifically [35–39,42,48]. Matching staff and service users by ethnicity, cultural backgrounds or religious views was extensively discussed [28,29,35,37,40,48,50,51,56]. Cultural and ethnic matching was investigated by two studies, revealing differing views among service users, leading to the provision of a choice in the studies [40,55]. One study with Jewish communities [55] considered language adopting terms such as “well-being” and “self-improvement” rather than anxiety or depression which are less accepted by these communities. Another study [32] employed positive language, referring to participants as “learners” to encourage engagement, rather than emphasising low literacy levels. Studies also discussed aligning interventions with existing community groups, considering religious and faith barriers such as dates of religious events [31,32,55].

Availability and accommodation, the third domain in the framework [14] was identified with six themes supported by 22 sources (flexible appointments, local spaces utilised, childcare facilities provided, transport provided, intervention delivered online and home visits – including delivery at an immigration centre). This domain pertains to the physical accessibility of health services, including geographic location, timing, appointment duration, and flexibility concerning work or childcare. Local community spaces such as places of worship, community centres and local parks were used in multiple studies [32,33,35,36,41,44,47,51,54]. Three studies discussed having flexible appointments to meet patient preferences such as work and family commitments [32–34]. Home visits were completed by three studies: one looked at dental care of Chinese undocumented migrant mothers [37], another exploring postnatal depression [38] and the final one provided a specialist maternity service to migrant mothers in an immigration accommodation centre [39]. Three studies delivered interventions in a community centre with childcare services [38,42,43]. A diabetes intervention was delivered online to African Caribbean populations [57,59] and another study designed a text messaging intervention of diet and activity advice [61].

The fourth domain of the framework is affordability [14], which relates to people’s ability to spend money, resources and time accessing health services. This domain identified 10 themes supported by 16 sources (flexible appointments, local community spaces, childcare facilities, local community spaces used, intervention in initial accommodation centre, intervention delivered online, home visits). Direct overhead costs such as transportation was discussed in four studies [32,38,39,43]. Travel costs were eliminated by utilising home visits in two studies [37,38]. Location of interventions was rarely discussed with regards to affordability for participants however, many studies utilised community spaces which may have indirectly supported affordability. There was minimal discussion of indirect participant costs. Three studies discussed how childcare was provided in the same community centre as the intervention [38,42,43]. Two studies reported the interventions were free [32,35] or free for the first six months [32]. One intervention was delivered online which was categorised in affordability [57,59].

Appropriateness, the fifth domain [14], is the fit between services and client needs, such as timeliness and care given to assess health problems and determine correct treatment. This domain identified 10 themes supported by 20 sources (cultural training of staff, multilingual facilitators, consideration of religious beliefs, ethnically matched staff, personalised care, self-referral, joining up of services, intervention provided after health event and use of interpreters). Staff cultural training was discussed by five studies [30–32,34,56] which included for example appropriate body language and culturally appropriate dress [32]. Two studies directly discussed the use of interpreters [39,51]. Six studies discussed the use of multilingual facilitators [27,32,36,38,42,50]. Four studies addressed low literacy levels by delivering interventions through pictures and/or verbal means [32,36,49,58]. Two studies delivered tailored or personalised care [37,40].

The thematic analysis identified three themes that did not fit into the patient-centred access framework, supported by four sources. These included public engagement, longevity of programmes and community involvement in planning healthcare interventions [31–34,47]. Dyson et al. [34] discussed an intervention with multiple components which supported each other and therefore success relied on all components being incorporated. Another study discussed the longevity of programmes, since short term interventions do not support ethnically underrepresented communities long term [32]. This is especially relevant considering many interventions targeted long-term conditions such as diabetes. Public engagement and community involvement in planning and delivering the intervention was discussed by several studies due to its importance in developing relationships and ensuring relevance to the target population [30–32,34,41,47].

## Discussion

### Main findings

Healthcare access and engagement for ethnically underrepresented communities is a complex and multifactorial issue requiring complex interventions. This scoping review synthesised the published literature and mapped intervention components to the five domains of the patient-centred access framework. Overall, 35 studies were identified which were predominately based in primary care/mental health services with interventions more frequently addressing approachability and acceptability from the patient-centred access framework with affordability and appropriateness less frequently addressed. The evidence was based on inclusive samples with equity-based factors limited in eligibility criteria.

### Comparison to other research

Interestingly, a scoping review of international studies exploring access and engagement [12] found that the majority of the included interventions targeted approachability, availability and affordability, in contrast to this review, which found that approachability and acceptability were the domains supported by most sources. This perhaps may relate to the fact that USA healthcare is not free at point of access in comparison to the NHS, altering the needs of non-UK populations. International studies discuss designated staff for outreach-based roles, including care co-ordinators and integrating teams, such as linking primary and secondary care for patients receiving care from multiple services [12]. Another scoping review [64] explored the role of community care navigators in Canada and whilst it found supporting evidence, only USA literature was identified. The role of care coordinators has not featured in UK literature in this review and may be a worthwhile focus of future research. This could be especially pertinent as early onset multi-morbidity has been found to be more prevalent in ethnically underrepresented communities in the UK compared to White British groups [65]. Many of the international studies [12] had larger sample sizes than UK studies included in this review. For example, one study explored creating a managed care plan for USA uninsured patients and had a sample size of 23,143 people [66]. This perhaps identifies that UK literature is in its infancy in comparison to USA literature of complex interventions to support health equity.

Systematic literature reviews in this area are dominated by studies completed in USA, despite being completed by authors in other high-income countries with publicly funded healthcare. A review of interventions for care-seeking behaviour in migrants by Swedish authors identified 16 studies – one Canadian and the rest USA [67]. They identified promise with community-based interventions but concluded that further research using robust study designs is required. Similarly, a review of Indigenous communities in USA and Australia identified lack of effectiveness assessment and called for more rigorous study designs [68]. However, a Canadian review of barriers and strategies for Indigenous communities identified the following strategies: financial support, education and employment, culturally sensitive information and systems and involvement of Indigenous communities and elders in policy processes [69]. A review of cultural adaptations for behaviour change (smoking, diet, physical activity) from the Netherlands identified 17 studies, all USA [70]. Another review of interventions for culturally and linguistically diverse populations included 24 studies (19 USA and one each in Canada, UK, Australia, Switzerland, Mexico) concluded that culturally trained bi-lingual health workers should be considered as part of potential solutions [71]. A systematic review from Finland identified Nordic studies but the review was focused specifically on cultural competence and outcomes were aimed at staff confidence/competence rather than that patient experience or outcomes [72].

In recognition of the lack of high-quality research in this area a Delphi study was completed in Denmark recommending more co-designed interventions, culturally and linguistically appropriate services, and policies targeted towards health literacy, linguistic, cultural and social differences [73]. Other literature (not reviews) includes a qualitative project looking at dementia in Norway which concludes that inclusive policies to promote patient centred approaches in ethnically underrepresented communities are required for appropriate dementia care [74]. A culturally tailored dementia education programme in Denmark also demonstrated success, however they did not assess long term knowledge post intervention [75].

### Interpretation

This review has demonstrated that complex interventions have been applied through multiple healthcare services, targeting ethnically underrepresented communities and have a key role to play in health inequalities. However, it is challenging to draw conclusions about the interventions included in this review due to effectiveness only being assessed in 12/35 studies (34%). Whilst the 34% that describe effectiveness mostly describe positive results (with few describing neutral results and none reporting negative results), most studies in this review do not examine how effective their interventions are. This is especially challenging when it is understood that whilst interventions aim to reduce inequalities, they also have the potential to generate them [76]. In a review of intervention generated inequalities in primary care, Gkiouleka and colleagues found a lack of robust evidence to investigate the outcomes of complex interventions [76]. They concluded that there are five factors that interventions in primary care should consider: being connected, intersectional, flexible, inclusive and community centred [76]. Many of these factors are echoed in the components of interventions included in this review supporting the role of complex interventions in helping to improve health equity.

Health inequalities arise from multiple intersecting factors beyond socioeconomic status (e.g., gender and ethnicity) and are shaped by structural elements such as education systems, political frameworks, housing policies and power imbalances [77]. Therefore, interventions aiming to reduce these inequalities require multifaceted components that are designed to target these intricate elements in particular groups, considering the complex interplay of factors (intersectionality). This is reflected in cultural adaptation of programmes, featured in several studies in this review. For example, interventions demonstrated positive effectiveness and included cultural adaptations for 1^st^ generation Bangladeshi women [36], South Asians with postnatal depression [38], South Asians with asthma [45], Black African, Caribbean or Black British people with diabetes [33,57,58] and ethnic minority women broadly [48]. Acceptability of cultural adaptations is also supported by studies in this review, further supporting the role of adapting services or programmes [40,57]. One significant challenge of “cultural adaptations” of interventions is the often limited detail in reporting of how these adaptations are completed, lacking information about how the adaptations make the intervention more “culturally acceptable”.

This limits potential learning from studies, reproducibility and application into future intervention development.

It also is important to consider the wider political and power structures that contribute or drive health inequalities and the impact that this has on communities. These wider power structures give a background to the importance of using community centred approaches to build trust and relationships. Trust, therapeutic alliance and relationship building were directly discussed by five articles in this review [30–32,47,55]. However, utilising community links, community ambassadors or outreach was used by 14 studies [28,32,33,41,42,44,47,51–55,58,61] and demonstrates the importance that many interventions place on working *with* target communities. Edge and colleagues were the only study describing relationship building that evaluated their intervention effectiveness [31]. They demonstrated a positive effect of their culturally adapted family intervention for Black African Caribbean people with schizophrenia [31]. Participatory methods were also utilised by several studies in this review, perhaps a methodology chosen to help to build trust and devolve power through the study process by involving target populations [33,34,47]. Whilst the importance of trust and relationship building was stressed [32], the matter of *how* to build trust was not reported on in detail. This leaves the question: how do we build trust and therapeutic alliance with ethnically underrepresented communities? And does this need to be approached differently for different ethnically underrepresented communities? Trust building with communities may also be challenging to research as it may involve activities that are difficult to document and evaluate. In their review, Cu et al. [78] also found that numerous studies lacked details on trust-building and engaging service users. Building trust is directly linked to the longevity of interventions. This is especially challenging with long term conditions such as diabetes which will involve lifelong behaviour and lifestyle changes. However, few projects evaluated outcomes beyond the short-term. It is therefore uncertain whether the interventions effectively ensure the trust built is preserved in the longer term. This challenge was highlighted by Such and colleagues who identified several short-term physical activity interventions but also highlighted how the three interventions they used as case studies also all aimed to build capacity to support long term delivery of interventions in the community [32].

Since only 12/35 studies assessed effectiveness, the potential for evidence-based adaptation is limited. Therefore, participation of target populations in intervention designs or adaptations becomes even more important to ensure that interventions will meet the needs of the communities they aim to serve. This highlights the further importance of participatory methodologies such as co-design to involve service users within intervention design and adaptation. Three studies included in this review utilised participatory methodologies [32,48,55], however 14 studies utilised community links, ambassadors or outreach [28,32,33,41,42,44,47,51–55,58,61]; highlighting the importance of participation within intervention (and research study) design and development.

It is well documented that structural systems of power contribute towards health inequalities [79,80]. Racism is pervasive and embedded within structures of society [81]. Structural racism is normalised and is embedded within societal norms, laws, cultures and institutions [80,82]. The Institute of Health Equity [79] highlights racism as having three key contributions towards poorer health over the life course. Firstly, racism directly damages physical and mental health. Secondly, racism may be linked to socioeconomic disadvantage and negative effects of social determinants of health. The third way, most relevant to this review, racism damages health through the operation of systems including health care system. Given this damaging effect that healthcare systems themselves can have on health inequalities it is a significant gap that none of the literature identified in this review of ethnically underrepresented communities mentions or discusses racism. The absence of discussion about racism across the included studies is especially striking given that many were authored by individuals working within institutions that both shape and sustain health inequalities [82]. Power often exists invisibly to those who hold it but is felt acutely by those who lack it; future research must make power visible and critically examine how it influences both research and service design itself.

The patient-centred access framework [14] provided an excellent framework for this scoping review. Selected for its comprehensive analysis of access and engagement in health services, this framework is commonly employed in studies focusing on underrepresented groups’ access [12,83,84]. However, some factors are not included in this framework and were not identified in the inductive thematic analysis that was independent of the framework. This includes the issue of power dynamics, an important factor when considering research and engagement with underrepresented communities and those with issues trusting research and/or healthcare. Interventions may more effectively address power imbalances by collaborating with communities through engagement activities and participatory methods, which may require effort to support engagement [2]. An Australian group discuss in detail the steps that they took to optimise engagement of ethnically underrepresented communities in the co-design process of two of their cancer trials which included inviting seldom heard voices and providing adequate resources [85]. Considering power dynamics is especially pertinent when considering the structural and systemic causes of health inequalities. Whilst the studies utilising participatory methodology will have considered the role of power within their research design, other studies did not discuss this. For example, Flanagan and colleagues provided an incentive to doctors in primary care to increase recruitment of “migrant” patients to viral hepatitis screening [60]. Whilst this study did see an increased uptake in participating GP practices this intervention fails to address the complex factors surrounding health inequalities such as barriers to accessing healthcare, discriminatory practices, or policies that marginalise specific groups [77]. More holistic approaches should be considered such as the complex intervention (HEAL-D) developed through extensive community engagement and participatory methods for Black African, Caribbean or Black British people with Diabetes. This intervention included components across all five of the patient centred access framework domains [33]; was shown to be highly acceptable [33], and to significantly improve participant confidence in controlling their diabetes and managing their diet in the adapted online version.

Understanding which ethnically underrepresented communities were targeted by these studies helps to appreciate how generalisable the results may be to other groups and communities. Overall, the studies employed wide eligibility criteria for participants. Of note was the specific nature of eligibility criteria which is seen less frequently in the wider evidence. For example, ethnicity determined by place of birth (first generation Bangladeshi) or heritage (South Asian). This is worth considering alongside the differences in intervention effectiveness between ethnic groups or generation of immigration in two studies in this review [45,58]. Moore and colleagues reported a better response in African Caribbean participants, which is logical considering that the cooking intervention utilised specific African Caribbean recipes so may have been less relevant to those from other cultural backgrounds [58]. However, it is curious that Griffiths and colleagues’ report the best results in 1^st^ generation South Asian immigrants in the intervention group, followed by the same population in the control group and a poorer response in higher generation immigrants in the intervention group [45]. Unfortunately, the study does not explore why the authors think this response was seen and therefore future studies may benefit from further exploring intervention responses between generations of migrants.

Culturally tailoring or adapting services has shown promise when applied to specific ethnically underrepresented communities and could be applied for example in service evaluation projects.. Whilst this review has highlighted key strategies for supporting ethnically underrepresented communities, only one third of studies have assessed the effectiveness of their interventions, limiting the impact of any conclusions drawn from these studies.

### Policy recommendations

This review has highlighted several areas for policymakers to consider in improving access to and engagement with health services for ethnically underrepresented communities. Central to this is the need to recognise and address power imbalances, as policies that overlook structural inequities risk reinforcing the very systems of exclusion they aim to dismantle. Making power visible – by engaging underrepresented communities in decision making processes, supporting the devolution of power, and applying structural analysis – can help transform the conditions that perpetuate unequal access. Aligned with this, trust emerged as a key theme across several studies in this review [33,34,47], underscoring the importance of working collaboratively with underrepresented communities through establishing community links. Building trust and tailoring policies to the cultural context of underrepresented communities is essential as culturally adapted approaches have been highlighted in this review as a promising way of supporting ethnically underrepresented communities. As this review focused on literature and interventions specifically within the UK, policy recommendations are also focused on the UK setting. However, there may be elements that may be relevant to other high-income countries with public health systems. For example, these recommendations align with existing work within Canada and Australia, partnering with Indigenous communities to share power in developing research and policy priorities [86,87].

### Future research

Future complex interventions in ethnically underrepresented communities should endeavour to assess the effectiveness of their work to provide conclusive results of strategies with ethnically underrepresented communities. Most of the studies in this scoping review were in mental health or primary care and therefore studies in other health services are needed. This is especially important for health services that may have specific cultural barriers, such as physiotherapy which requires enhanced engagement for success. Addressing the intricate challenges in healthcare access may necessitate multifaceted interventions covering all five domains of the patient-centred access framework. Particularly, future efforts should focus on enhancing affordability and appropriateness, identified as the least addressed components in this scoping review. Further, identified studies explored service-based interventions; it may be important for future studies to consider patients’ abilities to: perceive, seek, reach, pay and engage [14].

UK based interventions may also benefit from methods used in non-UK studies, such as care-coordinators and streamlining services. Future clinical research may support this approach due to the higher prevalence of early multimorbidity in ethnically underrepresented communities, and therefore the likelihood of those communities’ increased needs for multiple services.

Future research could make more efforts to increase visibility of power. This could be achieved by using positionality/reflexivity statements in outputs, adopting anti-racism approaches/interventions [88] and incorporating frameworks for structural analysis such as intersectionality [89] or critical race theory. Centring underrepresented voices through engaging with and compensating underrepresented communities for their contributions are other possible strategies. Other strategies include using participatory methodologies and developing opportunities for underrepresented communities to take positions of power within projects, for example as co-researchers [90]. Another approach is providing contextualised data by reporting on language, ethnicity and other intersecting factors. Ensuring that studies explore experiences and not just lack of engagement will provide further context to help understand the complexity and nuance of the challenges faced by underserved groups. Providing fair compensation for community contribution and redistributing power through providing opportunities for communities and underrepresented groups to take positions of power within projects.

### Limitations

Whilst this scoping review attempted to mitigate limitations by providing a clear and transparent audit process of the search strategy and analysis, some limitations remain. Whilst six electronic databases, reference lists and citations were searched, grey literature sources were not searched due to resources. The six databases chosen for this search were selected due to their strong coverage of interdisciplinary areas and to be inclusive of psychology literature which is a known area with relevant research to this topic. Following searches, reference lists were reviewed alongside citation tracking for further identification of relevant literature. However, it is acknowledged as a limitation that other relevant evidence indexed in other non-overlapping databases such as PubMed may not have been included. Searches were limited to the years 2014–2024. This is in line with the timings of the previous NHS long-term plan, “NHS Five year Forward View” [91], and with UK worsening inequalities in the last 10 years [92]. The time limit also aligns with UK population distribution changes and an increase in ethnically underrepresented communities between the 2011 and 2021 UK census [93]. However, it may have led to an underestimation of the relevant evidence.

This scoping review focused on ethnically underrepresented communities. However, it is recognised that factors such as socioeconomic status or religious beliefs, can also impact access and engagement directly or in intersection with ethnicity. Ethnic origin was selected for its common usage in healthcare access literature [8,10] and enhanced accessibility in UK electronic health records compared to other factors [93]. This review used the term “ethnically underrepresented communities” to acknowledge the structural inequalities in representation and access, with the aim to be a more inclusive and context-sensitive term than “ethnic minority”, which may imply inferiority and overlook systematic exclusion. While the growing use of the term “global majority” as a powerful reframing is recognised, it is not yet widely adopted in academic literature and “ethnically underrepresented communities” better reflects both the focus of this study and the realities of marginalisation within specific institutional and geographical contexts.

Only 10% of the themes were independently charted and cross checked by two researchers in the team, which can lead to subjectivity [78]. This is particularly relevant for components, such as the use of multilingual facilitators which were classified as appropriateness but arguably could be classified as availability or acceptability. The PROGRESS-Plus framework, endorsed by Cochrane whilst providing a lens to analyse the inclusivity of research studies, has also received some criticism for disregarding complexity and failing to incorporate intersectionality [94].

## Conclusion

Equitable healthcare access and engagement for ethnically underrepresented communities is a paramount goal of UK health services and public health. Despite existing research, only 35 studies met the inclusion criteria of this scoping review. These identified studies successfully implemented complex interventions to support equitable healthcare, especially in primary care and mental health. This review has shown that complex interventions may support tackling the social injustice of healthcare disparities and should consider all five domains of the patient-centred access framework. Future research should also extend scope beyond primary care and mental health services.

## Supporting information

S1 Appendix Keywords using PCC framework. (DOCX)

S2 Appendix Themes and codes. (DOCX)

S3 Appendix PROGRESS-Plus analysis. (DOCX)

S4 Appendix Categorisation of themes. (DOCX)

S5 Appendix PRISMA-Scr checklist. (DOCX)
