# Supplementary material for: Health service access for ethnically underrepresented communities: A scoping review of complex interventions
Source: PLoS One. 2026 Jan 6;21(1):e0340079. doi: 10.1371/journal.pone.0340079 (PMC12773815; doi:10.1371/journal.pone.0340079)
Supplement: S1 Appendix — (DOCX) [file pone.0340079.s001.docx]

## Appendix 1.0 Keywords using PCC framework

(adapted from Pollock et al., 2023)

| **PCC element** | **Keywords** | **Search terms and synonyms** | **Search strategy string** |
| --- | --- | --- | --- |
| **Population** | Ethnic minority groups | Ethnic minority group/racial minority group/Black asian and minority ethnic group | TI ("ethnic minorit*" or "Racial minorit*" or "minority ethnic grou*" or underserved or “black asian and minority ethnic group” or BAME or BME) OR  AB ("ethnic minorit*" or "Racial minorit*" or "minority ethnic grou*" or underserved or “black asian and minority ethnic group” or BAME or BME).ti,ab. |
| **Concept** | Interventions to improve access or equality | Intervention/access/equality | TI (Intervention* OR access OR inequalit* OR disparit* OR equalit*)  OR  AB (Intervention* OR access OR inequalit* OR disparit* OR equalit*).ti,ab. |
| **Context** | Within the National Health Service | National Health Service/NHS/health services | TI (NHS OR health OR “health service*” OR “health care”)  OR  AB (NHS OR health OR “health service*” OR “health care”).ti,ab. |
|  | Within the  United Kingdom | United Kingdom/England/Wales/Scotland/Ireland/Northern Ireland | TI (UK OR England OR Scotland OR Wales OR Ireland OR “Northern Ireland” OR “United Kingdom” OR “Great Britain”)  OR  AB (UK OR England OR Scotland OR Wales OR Ireland OR “Northern Ireland” OR “United Kingdom” OR “Great Britain”).ti,ab. |

**Cochrane search terms:**

(UK OR England OR Scotland OR Wales OR Ireland OR Northern Ireland OR United Kingdom OR Great Britain) AND (NHS OR health OR health NEXT service* OR health NEXT care) AND (Intervention* OR access Or Inequalit* OR disaprit* OR equalit*) AND (ethnic NEXT minorit* OR Racial NEXT Minorit* OR minority NEXT ethnic NEXT grou* OR underserved OR black asian and minority ethnic group OR BAME OR BME)

**PeDro (Physiotherapy database) keywords:**

Individual keyword combinations were utilised: "ethnic minorit* intervention" and "racial minorit* access"
