# Supplementary material for: Health service access for ethnically underrepresented communities: A scoping review of complex interventions
Source: PLoS One. 2026 Jan 6;21(1):e0340079. doi: 10.1371/journal.pone.0340079 (PMC12773815; doi:10.1371/journal.pone.0340079)
Supplement: S2 Appendix — (DOCX) [file pone.0340079.s002.docx]

## Appendix 2.0 themes and codes

| **Themes** | **Categories** | **Codes** |
| --- | --- | --- |
| 1. Approachability |  |  |
|  | 1.1 Outreach | 1.1.1 Outreach with designated roles to target populations e.g. support groups, Asian shops, Asian bridal show, yoga groups, English language courses |
|  |  | 1.1.2 Outreach to places of worship including faith leaders, community groups and organisations and building community links |
|  |  | 1.1.3 Community champions or ambassadors developed to build community links and identify targets for programme |
|  | 1.2 Information | 1.2.1 Education about what services are available (health services and charities) |
|  |  | 1.2.2 Flyers and adverts in community centres |
|  |  | 1.2.3 Adverts in community spaces and on social media |
|  |  | 1.2.4 Communication via text messages, phone calls and letters |
|  | 1.3 Screening | 1.3.1 Screening of patients through primary care, urgent care or emergency departments |
| 1. Acceptability | 2.1 Cultural values and norms | 2.1.1 Cultural adaptations of programmes especially considering collectivism and social support (involving families, family, community structures and community leaders) |
|  |  | 2.1.2 Building trust and relationships with communities |
|  |  | 2.1.3 Culturally or ethnically matched trained therapists delivering interventions |
|  |  | 2.1.4 Using religious spaces to increase cultural and social acceptability |
|  |  | 2.1.5 Changing focus of programme such as professionals focusing on quality of life rather than eliminating symptoms |
|  |  | 2.1.6 Incorporating cultural and religious values into interventions including dates of religious events |
|  |  | 2.1.7 Adaptation of language to be more positive such as wellbeing rather than anxiety and learners instead of low literacy levels |
|  | 2.2 social acceptability | 2.2.1 Familiar staff used in the programmes |
|  |  | 2.2.2 Incorporating social support and social comparison into interventions |
|  |  | 2.2.3 Activities with minimal social and cultural barriers such as walking groups |
|  | 2.3 literacy acceptability | 2.3.1 Engagement/information film made with community stakeholders |
|  |  | 2.3.2 Content verified by native speakers/ethnic matching |
|  |  | 2.3.3 Consideration of literacy level of participants |
|  | 2.4 Gender values and norms | 2.4.1 Gender split sessions e.g. women’s only sessions |
| 1. Availability and accommodation | 3.1 Geographic location | 3.1.1 Utilising religious spaces to increase convenience |
|  |  | 3.1.2 Local community spaces used (near places of worship or using local parks) |
|  | 3.2 Accommodation | 3.2.1 Home visits (including delivery in an immigration centre) |
|  |  | 3.2.2 Intervention delivered in a community centre with childcare facilities |
|  | 3.2 Hours of opening | 3.2.1 Flexibility of appointments made around patient preferences (to suit family and work commitments) |
|  |  | 3.2.2 Weekly sessions scheduled around patient preferences |
|  | 3.3 Appointment mechanisms | 3.3.1 Booking reminder systems e.g. text messages |
| 1. Affordability | 4.1 Direct costs | 4.1.1 Intervention sessions offered free of charge to participants |
|  |  | 4.1.2 Childcare offered in the same community centre the intervention is delivered in |
|  |  | 4.1.2 Overhead costs such as travel provided by the programme |
|  |  | 4.1.3 Home visits reduce the necessity for patient travel costs |
|  |  | 4.1.4 Intervention delivered online (or via phone) |
|  |  | 4.1.5 Participants reimbursed for attendance in study |
|  | 4.2 Indirect costs | 4.2.1 Protected funding for outreach workers |
|  |  | 4.2.2 Time off work required due to appointments being in work hours |
|  | 4.3 Opportunity costs | Not discussed |
| 1. Appropriateness | 5.1 Technical and Interpersonal quality | 5.1.1 Literacy levels considered in planning and delivery of programme by using verbal or pictorial information |
|  |  | 5.1.2 Cultural training for staff delivering the intervention |
|  | 5.2 Adequacy | 5.2.1 Multilingual staff/facilitators |
|  |  | 5.2.2 Interpreters to assist intervention and other required activities |
|  |  | 5.2.3 Patient choice if staff delivering the intervention are ethnically or religiously matched (due to being advocated by some individuals and not by others) |
|  | 5.3 Coordination and continuity | 5.3.1 Personalised care |
|  |  | 5.3.2 Self-referral |
|  |  | 5.3.3 Supported the joining up of services and signposting |
|  |  | 5.3.4 Continuity of staff (e.g. same staff members delivering the intervention) |
| 1. Additional themes outside the patient centred access framework | 6.1 Patient and Public Involvement | 6.1.1 Patient and Public Involvement in designing and planning the interventions |
|  | 6.2 programme length | 6.2.1 Longevity of programmes |
|  | 6.3 Intervention design | 6.3.1 Multicomponent interventions |
