# Supplementary material for: Health service access for ethnically underrepresented communities: A scoping review of complex interventions
Source: PLoS One. 2026 Jan 6;21(1):e0340079. doi: 10.1371/journal.pone.0340079 (PMC12773815; doi:10.1371/journal.pone.0340079)
Supplement: S3 Appendix — (DOCX) [file pone.0340079.s003.docx]

## Appendix 3.0 PROGRESS-Plus analysis

|  | **Inclusion criteria** | **Exclusion criteria** | **Baseline characteristics** | **Subgroup analyses** |
| --- | --- | --- | --- | --- |
| **Place of residence** | Initial accommodation centre (56) | Care home residents (44) | Housing rented, owned or shared (36) |  |
|  | Interviews completed in participants own home (37,43) | Temporary accommodation (46) |  |  |
| **Race/**  **ethnicity /culture** | Roma and Traveller communities (53) | Not 1^st^ generation South Asian immigrants (34,35) | Roma and Traveller communities (53) | Asian vs Black people heritage sign up for organ donation (24) |
|  | Minority ethnic groups broadly (23–25,28,47,51,52,55) | Not 1^st^ generation immigrant from Democratic Republic of Congo (46) | Black African, Caribbean or Black British (24,27,29,39–41,44,54) | Ethnicity, 1^st^ generation vs following generation immigrants (36) |
|  | “Migrants” (40,56) | Not African, Caribbean or Black British (23,44) | Asian Caribbean - Roche | African Caribbean ethnicities vs “other” ethnic group (39) |
|  | South Asian (34,36–38,48,49,55) |  | “Asian” (24) or South Asian (35,40,47,55) |  |
|  | Jewish communities (31) |  | Eastern European (40) |  |
|  |  |  | White British (47) |  |
|  | Born in the Democratic Republic of Congo (46) |  | "Other" (39,40) |  |
|  |  |  | Arab, Mixed Arab and Turkish (47) |  |
|  | Black African, Caribbean or Black British (27,29,39,41,54,57,60) |  | Vietnamese, Chinese, Albanina, Nigerian, Afhan, Yemeni (56) |  |
|  |  |  | Generation in the UK (1st, 2nd, 3rd, 4th) (36,37) |  |
| **Occupation** |  |  | Employment status (27,29,37,44) |  |
|  |  |  | Occupation (including carers and homemakers) (37,53,55) |  |
|  |  |  | Sick, disabled or in receipt of welfare benefits (39) |  |
| **Gender/sex** | Women (33,34,37,38,43,49,51,56) |  | Reported gender or sex (29,35,36,39,46) |  |
| **Religion** | Muslims (45) |  | Muslim (45,55) |  |
|  |  |  | Christian (27,55) |  |
| **Education** |  |  | Highest educational attainment (27,29,35,39,44) | Level of educational attainment (39) |
| **Time dependent characteristics** | Women eligible for breast screening (33) | Patients that had already taken part in hepatitis screening (40) | Settled in the UK for less than 3 years (43) |  |
|  | Post partum (49) |  |  |  |
|  | Utilised maternity service within the last 3 months (56) |  |  |  |
| **Age** | >16 (38,49) |  | Young adults (18 – 39 years) (27,37,40,47,54) | Age categories (36) |
|  | >18 (27,29,34,36,37,47,48) |  | Middle aged adults (40 – 64 years) (27,39–41,47,54) |  |
|  | >25 years (51) |  | Older adults (65 years +) (40,54) |  |
|  | >50 (44) |  |  |  |
| **Disability** | Post partum depression (38,47) | Severe physical or learning disability (37,38) | Marital status (37,43,55) |  |
|  | Type 2 diabetes (29,39,41,48,60) | Severe mental health problems including depression and psychosis (29,37,52) |  |  |
|  | Asthma (36) | Actively suicidal/high risk to self and others (27,36–38) |  |  |
|  | Depression (37,45) | Mobility difficulties preventing attendance (29) |  |  |
|  | Mental health diagnoses (31,42,47) | Chronic condition that would impact diet and activity behaviour (39)** |  |  |
|  | Schizophrenia (27) | Not diabetic (29) |  |  |
|  |  | “Complex clinical needs” (29) |  |  |
|  |  | Cognitive impairment, dementia diagnosis or lacking capacity to consent (27,44) |  |  |
| **Plus** |  | Pregnant (29) | Migrant status/residency (46,47) |  |
|  |  | Participating in another research study (29) | Country of birth (27,46) |  |
| **English fluency** | English fluency (36) | Not speaking English, Hindi, Urdu or Punjabi (27,35,39,48) |  | English fluency levels (36) |

Socioeconomic status and social capital not reported by any studies therefore omitted from table

* South Asian including Indian, Pakistani, Bangladeshi, other South Asian heritage

**Excluded as intervention included diet and activity components
