# Supplementary material for: Health service access for ethnically underrepresented communities: A scoping review of complex interventions
Source: PLoS One. 2026 Jan 6;21(1):e0340079. doi: 10.1371/journal.pone.0340079 (PMC12773815; doi:10.1371/journal.pone.0340079)
Supplement: S4 Appendix — (DOCX) [file pone.0340079.s004.docx]

## Appendix 4.0 categorisation of themes

| **Thematic domains** | **Category of domain** | **Supporting sources** | **% of** | **Study design applied** | **Health service settings** | **Period covered** |
| --- | --- | --- | --- | --- | --- | --- |
| 1. Approachability | Outreach with designated roles to target populations e.g. support groups, Asian shops, Asian bridal show, yoga groups, English language courses | 14  (23,24,29,31,35,47,52–55,58,60) | 40% | Intervention mapping, qualitative, feasibility, protocol, mixed methods service evaluation, co-design workshops | Primary care, community mental health | 2015 - 2023 |
|  | Outreach to places of worship including faith leaders, community groups and organisations and building community links | 5 (28,29,31,51,57) | 14% | Randomised control feasibility trial, qualitative study, analysis of a secondary dataset of interventions and intervention development using focus groups, mixed methods | Primary care, community mental health | 2014 - 2021 |
|  | Community champions/ambassadors to raise awareness and identify targets for programme | 6 (31,46,48,52,54,59) | 17% | Mixed methods, intervention development, qualitative, co-design | Community mental health, primary care | 2015- 2023 |
|  | Signposting, educating or referring to other available services (health services and charities) | 4 (26,27,43,54) | 11% | Protocol of an intervention and mixed methods feasibility study of development, implementation and acceptability, quasi-experimental with no randomisation, qualitative | Dental health, mental health | 2016 - 2019 |
|  | Advertisements in community spaces and social media | 5 (26–28,47,58) | 14% | Protocol, implementation and acceptability, analysis of a secondary dataset of interventions and intervention development, co-design | Community mental health, primary care | 2016 - 2022 |
|  | Communication via text messages, phone calls and letters | 5 (32,33,37,44,48) | 14% | Mixed methods feasibility study, intervention development, observational, cluster randomised control trial | Mental health (maternal, primary care | 2014 - 2023 |
|  | Screening of patients through primary care, urgent care or emergency departments | 9 (23,29,32,33,35,36,40,44,55) | 26% | Cluster randomised control trial, qualitative (focus groups) | Primary care | 2016 - 2019 |
| 1. Acceptability | Cultural adaptations of programmes | 10  (26,27,29,33,36,37,41,44,45,57) | 29% | Protocol of an intervention, qualitative study, mixed methods, mixed methods feasibility study, intervention development, qualitative, cluster randomised trial | Mental health and primary care | 2015 - 2024 |
|  | Building trust and relationships with communities | 5 (26–28,31,46) | 14% | Protocol of an intervention, mixed methods feasibility study and analysis of a secondary dataset of interventions, mixed methods including qualitative and co-design | Mental health and primary care | 2016 - 2023 |
|  | Culturally or ethnically matched staff (including peer outreach workers) | 8 (24,25,33,42,45,47,51,55) | 23% | Mixed methods study, qualitative study, co-design, observational | Primary care and mental health | 2014 - 2022 |
|  | Using religious spaces to increase cultural and social acceptability | 3 (28,37,58) | 9% | Analysis of a secondary dataset of interventions, mixed methods feasibility study and intervention development | Primary care and mental health | 2019 |
|  | Changing focus of programme such as professionals focusing on quality of life rather than eliminating symptoms | 3 (26,27,53) | 9% | Mixed methods, protocol and feasibility study | Primary care and community mental health | 2016 - 2020 |
|  | Incorporating cultural and religious values into interventions including dates of religious events | 3 (28,31,59) | 9% | Analysis of a secondary dataset of interventions, qualitative and mixed methods | Primary care and community mental health | 2015 - 2019 |
|  | Adaptation of language to be more positive such as wellbeing rather than anxiety and learners instead of low literacy levels | 2 (28,31) | 6% | Analysis of a secondary dataset of interventions, mixed methods | Primary care and community mental health | 2017 |
|  | Familiar staff used in the programmes | 2 (28,56) | 6% | Analysis of a secondary dataset of interventions and service evaluation | Primary care and maternity care | 2019 - 2020 |
|  | Incorporating social support and social comparison into interventions | 3 (28,37,57) | 9% | Analysis of a secondary dataset of interventions, mixed methods feasibility study and intervention development | Primary care and mental health | 2019 |
|  | Activities with minimal social and cultural barriers such as walking groups | 2 (28,48) | 6% | Analysis of a secondary dataset of interventions and intervention development | Primary care | 2017 - 2023 |
|  | Engagement/information film made with community stakeholders | 1 (35) | 3% | Prospective feasibility study | Primary care | 2020 |
|  | Content verified by native speakers/ethnic matching | 1 (39) | 3% | Feasibility (pre-test post-test) | Primary care | 2023 |
|  | Consideration of literacy level of participants | 1 (44) | 3% | Mixed methods: Qualitative and feasibility cluster randomised controlled trial | Primary care | 2018 |
|  | Gender split sessions | 3 (28,31,49) | 9% | Analysis of a secondary dataset of interventions and qualitative study nested in a randomised control trial, mixed methods | Primary care and mental health | 2015 - 2017 |
| 1. Availability and accommodation | Local community spaces such as local parks, community centres, places of worship, local parks | 11 (24,28,34,35,37,45–47,51,54,58) | 31% | Qualitative, pre-test post-test pilot study, Mixed methods, secondary analysis of dataset of interventions, co-design, prospective feasibility study | Primary care and mental health | 2014 - 2023 |
|  | Home visits (including delivery in an immigration centre) | 3 (37,43,56) | 9% | Quasi-experimental study, mixed methods feasibility study and qualitative, service evaluation | Dental care, maternity community mental health | 2019 - 2020 |
|  | Intervention delivered online | 3 (41,48,60) | 9% | Intervention development, protocol, feasibility study | Primary care | 2023 |
|  | Community centre with childcare facilities | 2 (37,38) | 6% | Mixed methods feasibility study and randomised control trial | Community mental health | 2019 - 2023 |
|  | Flexible appointments made around patient preferences (to fit family and work commitments) | 5 (26–29,53) | 14% | Intervention mapping with focus group, protocol of feasibility study, mixed methods feasibility study, analysis of a secondary dataset of interventions | Primary care and mental health | 2016 - 2021 |
| 1. Affordability | Sessions offered for free | 2 (28,51) | 6% | Qualitative and secondary data analysis of interventions | Primary care | 2014 - 2017 |
|  | Childcare facilities provided as part of intervention | 2 (37,50) | 6% | Mixed methods feasibility study and randomised control trial | Community mental health | 2019 - 2023 |
|  | Overhead costs such as travel provided | 4  (28,37,49,56) | 11% | Secondary analysis of an intervention dataset, mixed methods feasibility study, service evaluation, nested qualitative study | Primary care, community mental health and maternity care | 2015 - 2020 |
|  | Home visits | 3 (37,43) | 9% | Quasi-experimental study with no randomization, mixed methods feasibility and qualitative | Dental care and community mental health | 2019 - 2020 |
|  | Intervention delivered online (or via phone) | 3 (41,48,60) | 9% | Intervention development, protocol, mixed methods service evaluation | Primary care | 2022 - 2024 |
|  | Participants reimbursed for attendance in study | 3 (39,46,48) | 9% | Intervention development and mixed methods (qualitative and co-design), feasibility (pre-test, post-test) | Primary care | 2023 |
|  | Protected funding for outreach workers | 2 (23,53) | 6% | Qualitative and intervention mapping with focus groups | Primary care and community mental health | 2019 - 2020 |
|  | Timing of sessions to accommodate childcare duties | 1 (28) | 3% | Secondary analysis of an intervention dataset | Primary care | 2017 |
| 1. Appropriateness | Verbal or pictorial information given to accommodate low literacy level participants | 2 (28,34) | 6% | Pre-test post-test pilot study and secondary data analysis of an intervention dataset | Primary care | 2016 - 2017 |
|  | Cultural training of staff (including sensitivity of language, dress, faith practices etc. | 4 (26–28,42) | 11% | Secondary analysis of an intervention dataset, intervention mapping, protocol, and mixed methods | Primary care and mental health | 2016 - 2020 |
|  | Multilingual facilitators | 6 (23,28,34,37,49,55) | 17% | Secondary data analysis of an intervention dataset, pre-test post-test pilot study, mixed methods feasibility study and qualitative | Primary care and mental health | 2016 - 2019 |
|  | Use of interpreters | 2 (47,56) | 6% | Service evaluation project and co-design | Maternity care and primary care | 2020 - 2022 |
|  | Ethnically matched staff (or option provided) | 3 (31,43,45) | 9% | Quasi-experimental study, intervention development, mixed methods | Dental care and primary care | 2015 - 2019 |
|  | Personalised care | 3 (43,45) | 6% | Quasi-experimental study and qualitative | Dental care, community mental health | 2015 - 2019 |
|  | Self-referral | 1 (31) | 3% | Mixed methods | Community mental health | 2017 |
|  | Supported the joining up of services and signposting | 2 (31,54) | 6% | Mixed methods and qualitative | Community mental health | 2017 |
|  | Continuity of staff (e.g. same staff members delivering intervention or receptionists) | 2 (28,37) | 6% | Secondary analysis of an intervention dataset, feasbility study | Primary care and community mental health | 2017 - 2019 |
| 1. Themes outside of patient centres access framework | Public engagement and community involvement in planning and delivering the intervention | 6  (26–28,46,53,57) | 17% | Protocol of an intervention, mixed methods feasibility study and analysis of a secondary dataset of interventions | Mental health and primary care | 2016 - 2017 |
|  | Longevity of programmes | 1 (28) | 3% | Secondary analysis of an intervention dataset | Primary care | 2017 |
|  | Multicomponent interventions | 1 (53) | 3% | Intervention mapping with focus groups | Primary care | 2020 |
